# Alveolar Capillary Dysplasia with Misalignment of Pulmonary Veins (ACD/MPV): A Case Series

**DOI:** 10.1155/2013/327250

**Published:** 2013-01-08

**Authors:** Joana Miranda, Gustavo Rocha, Henrique Soares, Ana Vilan, Otília Brandão, Hercília Guimarães

**Affiliations:** ^1^Division of Neonatology, Hospital of São João, Faculty of Medicine, University of Porto, Porto, Portugal; ^2^Division of Neonatology, Hospital of São João, Piso 2, Alameda Professor Hernâni Monteiro, 4200-319 Porto, Portugal; ^3^Department of Pathology, Hospital of São João, Faculty of Medicine, University of Porto, Porto, Portugal

## Abstract

Alveolar capillary dysplasia with misalignment of pulmonary veins (ACD/MPV) is a rare, fatal, developmental lung disorder, which usually presents as persistent pulmonary hypertension of the newborn (PPHN) unresponsive to treatment. The authors present their own experience with three cases admitted during the last 15 years.

## 1. Introduction

Alveolar capillary dysplasia with misalignment of pulmonary veins (ACD/MPV; OMIM number 265380) is a rare, fatal, developmental lung disorder, which usually presents as persistent pulmonary hypertension of the newborn (PPHN) unresponsive to treatment [1, 2]. The majority of the reported cases have been associated with other multiple congenital nonlethal anomalies, most frequently involving the cardiovascular, gastrointestinal, urogenital, and musculoskeletal systems [1]. Increasing awareness of this clinical entity may prevent the use of more invasive and futile treatments, including extracorporeal membrane oxygenation (ECMO).

We present three cases of ACD/MPV associated with gastrointestinal and urological malformations. All the newborns had an overwhelming course, with PPHN and hypoxemia refractory to treatment. The diagnosis of ACD/MPV was established by autopsy.

## 2. Cases Report

### 2.1. Case Report 1

Full-term female newborn admitted to our NICU (Neonatal Intensive Care Unit) in 1997 was diagnosed with severe bilateral hydronephrosis. She was the second daughter of a young healthy, unrelated couple. On prenatal ultrasounds severe bilateral hydronephrosis and oligoamnios were detected. Delivery occurred by C section at 38-week gestational age. The Apgar score was 5/8 and birth weight 3170 g. During the first hours of life patient developed increasing respiratory distress, with hypoxemia and bradycardia, and was intubated and ventilated. Chest radiograph showed a mild haziness pattern and echocardiogram excluded structural heart disease and demonstrated signs of PPHN. At hour 30 of life a rapid clinical deterioration was observed, with refractory hypoxemia and persistent severe metabolic acidosis, despite ventilatory optimization. Antibiotic therapy with ampicillin and gentamicin was administered since admission to NICU and was later adjusted to ampicillin, cefotaxime, and amicacin. Septic workup was negative, including blood, urine, and cerebrospinal fluid cultures.

The planned renal ultrasound and voiding cystourethrography (VCUG) were not preformed since the patient's clinical condition deteriorated rapidly and newborn died on the second day of life.

On autopsy severe bilateral hydronephrosis was confirmed and signs of severe renal corticomedullary parenchyma atrophy were observed. Histologic evaluation of lung tissue showed the characteristic histological features of ACD/MPV.

### 2.2. Case Report 2

A neonate was admitted to our NICU during 2011 for surgical correction of duodenal atresia. She was a 36-week gestational age female newborn, of birth weight of 3800 g, and of a healthy 15-year-old primigravida. After an uneventful pregnancy, the delivery occurred by vacuum extraction and the newborn required resuscitation with oxygen mask. The Apgar score was 5/9/9. The newborn was submitted to an exploratory laparotomy which detected a duodenal stenosis secondary to annular pancreas. A surgical correction with duodenoduodenostomy was performed.

By hour 9 of life, a rapidly increasing respiratory distress syndrome developed and she was started on mechanical ventilation with FiO_2_ 1.0. After surgery, a clinical picture of PPHN with increasing oxygenation index became obvious, corroborated by echocardiographic findings. The patient's condition progressively deteriorated despite aggressive treatment for severe pulmonary hypertension, including inotropic support with dopamine, dobutamine and noradrenalin, inhaled nitric oxide (up to 40 ppm), oral sildenafil (maximum 2 mg/kg/day), and nebulized iloprost (maximum 3 *μ*g/kg/dose, 4/4 h). A diffuse reticulogranular pattern on chest radiographs became evident. Five doses of surfactant were administered. Infectious workup, metabolic screening, thyroid function, and cranial and abdominal ultrasounds were normal. Karyotype was 46,XX. The neonate presented an overwhelming course, with hypoxemia refractory to treatment, and died on day 15 of life.

**Figure 1:**
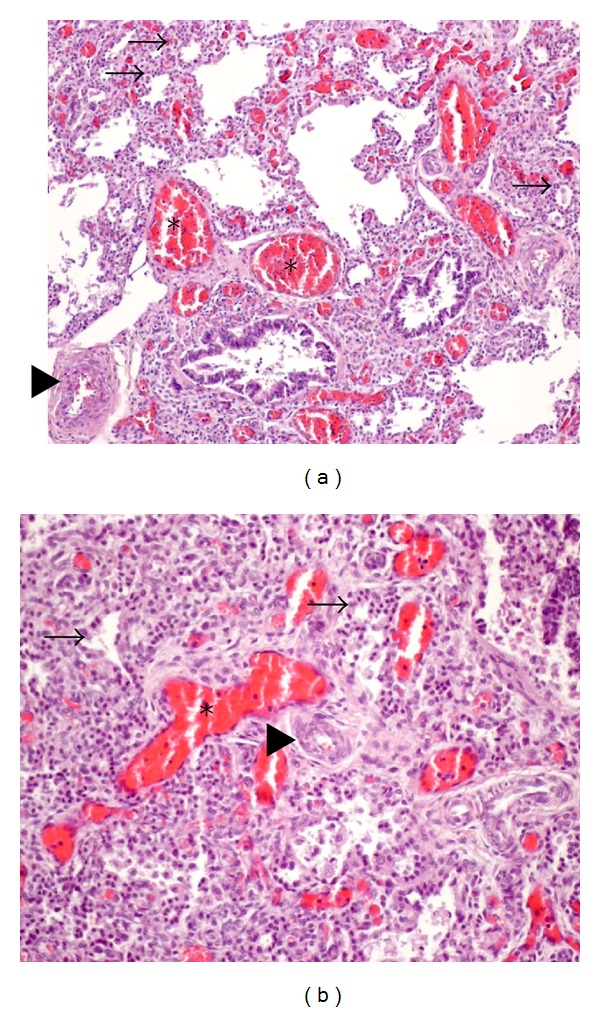
Case report 2: necropsy lung histology (hematoxylin and eosin staining) demonstrates the characteristic histologic features of ACD/MPV: thickened alveolar septae with scarce dilated pulmonary capillaries located away from the alveolar epithelium, with absence of the usual alveolar-capillary barrier (→); medial hypertrophy of small pulmonary arteries and muscularization of distal arterioles (▸); congested pulmonary veins malpositioned, adjacent to pulmonary arteries in the same adventitial sheath (∗); lymphangiectasis not present.

Autopsy confirmed the annular pancreas and revealed an associated intestinal malrotation. The lung histology was diagnostic of ACD/MPV (Figure 1).

DNA sequence analysis was performed, applying the protocol described by Sen and colleagues [3]. DNA sequence analysis revealed a heterozygous nonsense mutation c.539C>A, p.S180X, in the first exon of *FOXF1*. The mutation was not present in the mother. The father, an unrelated, healthy 21-year-old, refused testing.

### 2.3. Case Report 3

A male preterm newborn, of 34-week gestational age, with prenatal diagnosis of severe bilateral hydronephrosis associated with hydramnios, right pleural effusion, and ascites, was admitted to our NICU in 2012. Amniocentesis showed a normal karyotype (46, XY) and maternal serologic screening was negative. He was the first baby of a healthy young couple.

After a C-section delivery due to fetal distress, he presented with anasarca and respiratory distress, which improved temporarily with positive end-expiratory pressure during initial ventilation. The Apgar score was 2/8/8 and birth weight 2465 g. 

**Figure 2:**
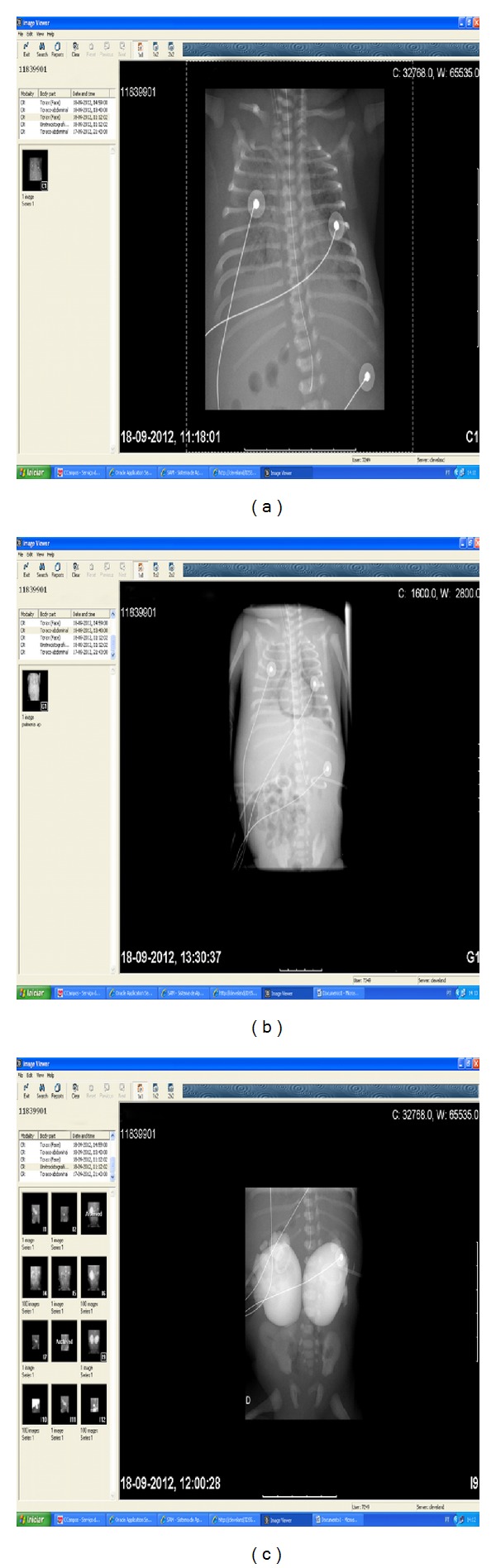
Case report 3: (a) chest radiograph with pulmonary ground glass opacities and right pleural effusion. (b) Chest radiograph showing pneumomediastinum. (c) Percutaneous nephrostomy using water-soluble contrast revealing very severe bilateral hydronephrosis.

During the first hour of life he was started on nasal CPAP, due to an increasing respiratory distress syndrome. Although, at hour 6 of life patient required intubation and mechanical ventilation owing to a persistent pulmonary hypertension, with increasing oxygenation index. Echocardiographic evaluation revealed a severe PPHN with a normal cardiac anatomy and chest radiographs showed ground glass opacities and right pleural effusion (Figure 2(a)). A rapidly increasing FiO_2_ to 1.0 was required and at hour 22 of life inhaled nitric oxide with 20 ppm was initiated, which was later increased to 40 ppm. Surfactant was administered twice. Nevertheless a severe hypoxemia refractory to treatment was settled. Cardiorespiratory arrest occurred on the second day of life, requiring respiratory and cardiac resuscitation with chest compressions and adrenaline. Pneumomediastinum with spontaneous resolution was observed, (Figure 2(b)). On the third day of life the newborn died after an unsuccessful trial of high-frequency oscillatory ventilation.

To investigate prenatal diagnosis of hydronephrosis a postnatal renal ultrasound and a VCUG were performed. The postnatal ultrasound revealed bilateral renal dilatation, with loss of corticomedullary differentiation, marked calyceal dilatation (renal pelvis anteroposterior diameter in the right kidney 35 mm and in the left kidney 25 mm), and ureteral dilatation. VCUG showed a bladder with an atypical morphology, elongated and irregular in its upper portion, but it was not suggestive of posterior urethral valves. To complement the study, an ultrasound-guided percutaneous nephrostomy using water-soluble contrast was preformed, revealing a very severe bilateral hydronephrosis with obstruction to contrast flow from the renal pelvis to the proximal ureter, suggestive of ureteropelvic junction obstruction, (Figure 2(c)). Bilateral percutaneous nephrostomies kept functioning until newborn's death.

Treatment with ampicillin and cefotaxime was initiated on admission to NICU. Blood and urine cultures were negative. The morphological study, including cranial and abdominal ultrasounds, was normal.

On autopsy the lungs were both heavy and globoid on gross appearance and the histopathologic study showed the characteristic features of ACD/MPV. Giant hydronephrosis secondary to ureteropelvic junction obstruction was evident. Renal corticomedullary parenchyma was normally developed, however with secondary atrophy and rare cortical tubules with cystic dilatation.

Due to the fulminate course of the disease, DNA was not extracted to further analysis and metabolic screening was not preformed. 

## 3. Discussion

Newborns affected with ACD/MPV develop respiratory distress and severe PPHN within the first 48 hours of life and die of respiratory failure within the first month of life, although longer survivals and later presentations have been reported [2, 4–6]. PPHN secondary to ACD/MPV may be difficult to distinguish from idiopathic PPHN. ACD/MPV should be suspected in patients with severe hypoxemia and PPHN who fail to improve with ventilatory support and pulmonary vasodilator therapy [2]. The clinical approach to infants with ACD/MPV is similar to other neonates presenting with PPHN, including inhaled nitric oxide, oral sildenafil, nebulized iloprost, and inotropic support with noradrenalin and milrinone. However, the response to this therapy is often minimal and not sustained, which may serve as an initial diagnostic clue. In some centres, ECMO is offered while awaiting definitive diagnosis. ACD/MPV should always be considered in infants with idiopathic PPHN without sustained response to ECMO [2]. 

Histological examination of lung remains the gold standard for ACD/MPV diagnosis. Thus, approximately 90% of reported cases of ACD/MPV have been diagnosed on autopsy and 10% of diagnoses have been made from lung tissue obtained during *ante mortem* lung biopsy [2]. Histologically, ACD/ MPV is characterized by malpositioned (misaligned) pulmonary veins, paucity of capillaries close to the alveolar epithelium, anomalous distended pulmonary veins within the bronchovascular bundle instead of the interlobular septa, and immature alveolar development with medial thickening of small pulmonary arteries [1, 2]. ACD/MPV is a rare developmental disorder of the lung affecting both the parenchyma and the vasculature [7]. 

More than 80% of infants with ACD/MPV have multiple other congenital malformations [1]. All of our newborns presented with other congenital malformations; two of them presented with severe bilateral hydronephrosis and the other with annular pancreas, and intestinal malrotation, anomalies already reported in literature [2, 8–10]. 

DNA sequencing and comparative genomic hybridization have led to the identification of *FOXF1* as one of the genes responsible for ACD/MPV. Using array CGH analysis, Stankiewicz and colleagues identified six overlapping microdeletions encompassing the *FOX* transcription factor gene cluster in chromosome 16q24.1q24.2 in patients with ACD/MPV and multiple congenital anomalies. Subsequently, they identified four different heterozygous mutations in unrelated patients with sporadic ACD/MPV and multiple congenital anomalies [11].

Using DNA sequencing analysis we identify a new heterozygous nonsense mutation in the first exon of *FOXF1* gene, c.539C>A, p.S180X in the patient with ACD/MPV, annular pancreas, and intestinal malrotation.

Unfortunately, in the two patients with ACD/MPV and severe bilateral hydronephrosis we did not have the opportunity to perform DNA sequencing analysis. Patients with ACD/MPV and hydronephrosis had already been described with mutations in the *FOXF1* gene by Stankiewicz and colleagues [11], as well as with 16q24.1 microdeletion in a case reported by Zufferey and colleagues [10]. 

In conclusion, our experience with ACD/MPV is according to the rare nature of the disease. There is no pathognomonic laboratory or imaging criteria for the disease. Initial chest radiographs are often reported to be unremarkable or to show a mild haziness pattern. The deteriorating course is a challenging task for diagnosis and treatment. Management of infants with ACD/MPV is not different than management of other causes of PPHN. Histological examination of lung remains the gold standard for ACD/MPV diagnosis. 

## Abbreviations

ACD/MPV: Alveolarcapillarydysplasiawithmisalignmentofpulmonaryveins
PPHN: Persistentpulmonaryhypertensionofthenewborn
NICU: Neonatalintensivecareunit
VCUG: Voidingcystourethrography
ECMO: Extracorporealmembraneoxygenation.
